# Subgenotype reclassification of genotype B hepatitis B virus

**DOI:** 10.1186/1471-230X-12-116

**Published:** 2012-08-27

**Authors:** Weifeng Shi, Chaodong Zhu, Wei Zheng, Michael J Carr, Desmond G Higgins, Zhong Zhang

**Affiliations:** 1 Guangzhou Institute of Advanced Technology, Chinese Academy of Sciences, Nansha, 511458, Guangzhou, China; 2 Key Laboratory of Zoological Systematics and Evolution, Institute of Zoology, Chinese Academy of Sciences, Beijing, 100101, China; 3 Shenzhen Institute of Advanced Technology, Chinese Academy of Sciences, Shenzhen, 518055, China; 4 Graduate University of Chinese Academy of Sciences, Beijing, 100049, China; 5 National Virus Reference Laboratory, University College Dublin, Dublin, 4, Ireland; 6 The Conway Institute of Biomolecular and Biomedical Research, University College Dublin, Dublin, 4, Ireland; 7 Department of Basic Medicine, Taishan Medical College, Taian, 271000, Shandong, China

**Keywords:** Hepatitis B virus, Subgenotype, Phylogenetic analysis, Sequence divergence

## Abstract

**Background:**

Nine subgenotypes from genotype B have been identified for hepatitis B virus (HBV). However, these subgenotypes were less conclusive as they were often designated based on a few representative strains. In addition, subgenotype B6 was designated twice for viruses of different origin.

**Methods:**

All complete genome sequences of genotype B HBV were phylogenetically analyzed. Sequence divergences between different potential subgenotypes were also assessed.

**Results:**

Both phylogenetic and sequence divergence analyses supported the designation of subgenotypes B1, B2, B4, and B6 (from Arctic). However, sequence divergences between previously designated B3, B5, B7, B8, B9 and another B6 (from China) were mostly less than 4%. In addition, subgenotype B3 did not form a monophyly.

**Conclusion:**

Current evidence failed to classify original B5, B7, B8, B9, and B6 (from China) as subgenotypes. Instead, they could be considered as a quasi-subgenotype B3 of Southeast Asian and Chinese origin. In addition, previously designated B6 (from Arctic) should be renamed as B5 for continuous numbering. This novel classification is well supported by both the phylogeny and sequence divergence of > 4%.

## Background

By comparing 18 HBV genomes, Okamoto et al. proposed that sequence divergence of > 8% over the entire genome should be used for HBV genotyping in 1988 [1]. Based on this, four genotypes were identified as A, B, C, and D. So far, at least eight genotypes, from A to H, have been identified and widely accepted. Genotypes B and C are prevalent in Asia and co-infection and/or super-infection lead to frequent recombination between these two genotypes [2,3].

Based on the rule that different subgenotypes should diverge by at least 4% over the genome [4], genotypes A [5], B [6], C [7], D [8], and F [9] have been reported to have evolved into various subgenotypes [10]. Genotype B was initially divided into two subgenotypes: Bj (j for Japan) and Ba (a for Asia) [2]. Viruses of subgenotype Bj are not recombinants, while those of subgenotype Ba are B/C recombinants, with their preC-C genes coming from genotype C [2]. In 2004, Bj was renamed as B1 and Ba was renamed as B2 [11]. In this report, subgenotypes B3 and B4 were also described. Subgenotype B3 was composed of four strains from Indonesia, while subgenotype B4 mostly comprised strains from Vietnam and France [11]. Subgenotype B5 was initially reported in 2006 from the Philippines [12]. A few months later, Sakamoto et al. found a few viruses from the Philippines that differed from subgenotypes B1 to B4, and also designated it as a novel subgenotype, B5 [13]. Subgenotype B6 was identified in 2007 from arctic indigenous populations [3]. In particular, just as with subgenotype B1, viruses of B6 were not recombinants. This observation led Sakamoto et al. to classify genotype B into two types, non-recombinant (B1 and B6) and recombinant (B2 to B5) [3]. Viruses of B7 were isolated from the Nusa Tenggara islands in Eastern Indonesia from 2008 [14]. Subsequently, subgenotype B8 was also identified in Indonesia by analyzing a large cohort of patient samples [15]. More recently, viruses of subgenotype B9 were isolated from the same region in Indonesia where subgenotype B8 was isolated [6]. This suggested that the distribution of HBV subgenotypes might relate to the ethnic origin of the infected patients [6].

However, previous studies just used a number of selected representative strains to perform phylogenetic analysis and estimate sequence divergence. Hence, the designation of novel HBV subgenotypes was less conclusive. In this study, we reanalyzed all of the full-length genome sequences of genotype B using phylogenetic analysis. This analysis led to the proposal of a novel and consistent classification for HBV of genotype B.

## Methods

We have previously analyzed a total of 3471 full length genome sequences of HBV using a phylogenetic approach [16]. Our results showed that 860 sequences belonged to genotype B. These sequences were selected to generate a new dataset for further analysis. In addition, a sequence of genotype C (GenBank:EU939604) was also included in the new dataset and used as an outgroup. Information of these sequences, such as subgenotype and recombination, was also extracted from GenBank annotations. For sequences with a reference available in Pubmed, we performed an extensive literature review to obtain their subgenotype and recombination information. This information was used to define the subgenotypes.

Phylogenetic analysis of the genotype B strains was carried out using RAxML [17] under the GTRCAT approximation [18] and random starting trees. Three thousand rapid bootstrap replicates were performed and all other parameters were set to default. Trees were visualized and analyzed using Dendroscope [19]. The tree is available as Additional file 1.

The mean nucleotide divergence (mean ± SD) between different subgenotypes was calculated using Mega 5 [20] under the Kimura 2-parameter model [21]. Five hundred bootstrap replicates were applied to obtain consistent and reliable sequence divergence values.

## Results

**Figure 1:**
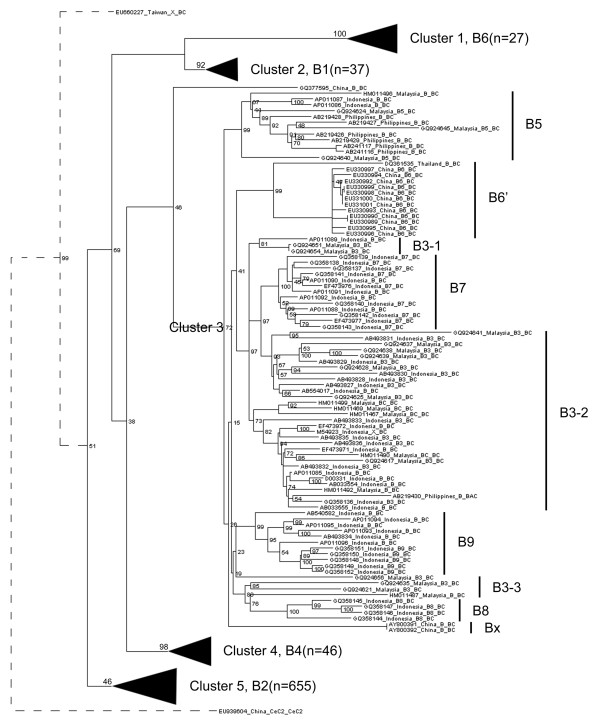
A schematic phylogenetic tree constructed using all genotype B HBV sequences.

Phylogenetic analysis of all HBV genotype B full-length genome sequences revealed five distinct major clusters. They were named as cluster 1 to 5 from top to bottom (Figure 1). Most of the clusters were well supported with high bootstrap values. Cluster 1 was composed of 27 viruses belonging to subgenotype B6 [3], with a bootstrap value of 100%. 37 viruses of cluster 2 were isolated from Japan and were designated as subgenotype B1 [2,11]. The bootstrap value for this cluster was 92%. 46 viruses of subgenotype B4 [11] constituted cluster 4, with a bootstrap value of 98%.

Phylogenetic analysis grouped 655 subgenotype B2 viruses into cluster 5 [2,11], representing approximately 76% of all genotype B strains. All viruses of this cluster were B/C recombinants, with the great majority of the strains isolated from Asia (Additional file 1). However, the bootstrap value for this cluster was only 46%.

Apart from the sequence divergence between B4 and B2 (3.9 ± 0.3%), sequence divergences between any other two subgenotypes (B6, B1, B4 and B2) were greater than 4% (Table 1). Furthermore, the within subgenotype divergences of these four subgenotypes were below 4% (Table 1).

**Table 1:** Mean nucleotide sequence divergences over the complete genome sequences of HBV between and within subgenotypes B6, B1, B4 and B2

**Subgenotypes**	**B6**	**B1**	**B4**	**Within group sequence divergence**
B6				0.027 ± 0.002
B1	0.062 ± 0.004			0.025 ± 0.001
B4	0.074 ± 0.005	0.055 ± 0.003		0.025 ± 0.001
B2	0.071 ± 0.004	0.047 ± 0.003	0.039 ± 0.003	0.017 ± 0.001

Cluster 3 was also well supported with a bootstrap value of 72%. Unlike the described clusters composed of a single subgenotype, cluster 3 included several previously reported subgenotypes, such as B5, B3, B9, B8 and B7 (Figure 1). In addition, a few Chinese strains named as B6 [22] were included into cluster 3. Due to the existence of another subgenotype B6 [3], the Chinese strains were tentatively renamed as B6’. Apart from the previously designated subgenotypes, two sequences isolated from China (GenBank:AY800391 and GenBank:AY800392) have not been designated a subgenotype and were tentatively named Bx. In particular, viruses of subgenotype B3 did not form a monophyly. Instead, subgenotype B3 viruses were scattered in three parts, B3-1, B3-2 and B3-3 (Figure 1).

To resolve the aforementioned problems, we calculated sequence divergences between B5, B3-1, B3-2, B3-3, B9, B8, B7, B6’ and Bx (Table 2). Apart from Bx, sequence divergence between any two of these potential subgenotypes was usually below 4% (Table 2). Although sequence divergences between Bx and other potential subgenotypes were mostly greater than 4%, the phylogeny did not support Bx to be a novel subgenotype in that it did not form a monophyly compared to other potential subgenotypes in this cluster (Figure 1). Therefore, sequence divergences did not support the designation of subgenotypes B5, B9, B8, B7, B6’, and Bx.

**Table 2:** Mean nucleotide sequence divergences over the complete genome sequences of HBV between previously designated subgenotypes in cluster 3

**Groups**	**B5**	**B6’**	**B3-1**	**B7**	**B3-2**	**B9**	**B3-3**	**B8**
B6’	0.039 ± 0.003							
B3-1	0.030 ± 0.002	0.028 ± 0.003						
B7	0.035 ± 0.002	0.033 ± 0.003	0.019 ± 0.002					
B3-2	0.037 ± 0.002	0.035 ± 0.003	0.023 ± 0.002	0.025 ± 0.002				
B9	0.033 ± 0.002	0.034 ± 0.003	0.025 ± 0.002	0.030 ± 0.002	0.032 ± 0.002			
B3-3	0.040 ± 0.002	0.040 ± 0.003	0.033 ± 0.002	0.037 ± 0.002	0.039 ± 0.002	0.037 ± 0.002		
B8	0.038 ± 0.002	0.038 ± 0.003	0.028 ± 0.002	0.033 ± 0.002	0.035 ± 0.002	0.033 ± 0.002	0.038 ± 0.002	
Bx	0.045 ± 0.003	0.046 ± 0.004	0.038 ± 0.003	0.042 ± 0.003	0.042 ± 0.003	0.042 ± 0.003	0.048 ± 0.003	0.045 ± 0.003

## Discussion

Previous studies have shown that different HBV genotypes and subgenotypes may cause differences in disease progression, response to anti-viral treatment regimens and in clinical outcomes [4,10,23]. Therefore, the accurate classification of HBV is important. To resolve the problems in HBV subgenotyping and to propose a consistent classification for genotype B HBV, we analyzed 860 complete genome sequences of genotype B using phylogenetic analysis.

Phylogenetic analysis showed that genotype B HBV has evolved into five major clusters. Four of them corresponded to B6 (cluster 1), B1 (cluster 2), B4 (cluster 4) and B2 (cluster 5). Apart from subgenotype B2, the remaining three subgenotypes were well supported by both high bootstrap values and sequence divergences of > 4%. Therefore, subgenotypes B6, B1 and B4 were properly designated. Although the bootstrap value for subgenotype B2 was only 46% and the sequence divergence between subgenotypes B4 and B2 was less than 4%, since subgenotype B2 has been widely accepted, it should be maintained in order to avoid more confusion.

However, our results failed to support the designation of subgenotypes B5, B3, B9, B8, B7 and B6’. First, subgenotype B3 was not a monophyly, but scattered in the tree. This was against the rule that an HBV genotype and subgenotype should be a monophyly [24]. Second, sequence divergences between the above subgenotypes were mostly less than 4%. This also did not support them to be separate subgenotypes.

Instead, considering that viruses in this cluster are all isolated from Southeast Asia and China and this cluster is well supported by a high bootstrap value of 72%, we proposed that a quasi-subgenotype B3 should be used for cluster 3 according to previous reports, in which the definition of quasi-subgenotype has been used to resolve the inconsistency in the subgenotyping of genotype A HBV [24,25]. Sequence divergences between the quasi-subgenotype B3 and B6, B1, B4 and B2 were 6.1 ± 0.4%, 6.8 ± 0.3%, 5.1 ± 0.3% and 5.1 ± 0.3%, respectively (Table 3). In addition, the within subgenotype divergence of quasi-subgenotype B3 was 3.1 ± 0.1%, less than 4% to differentiate a subgenotype.

**Table 3:** Mean nucleotide sequence divergences over the complete genome sequences of HBV between quasi-subgenotype B3 and other subgenotypes

	**newB5 (previous B6)**	**B1**	**B4**	**B2**
quasi-subgenotype B3	6.1 ± 0.4%	6.8 ± 0.3%	5.8 ± 0.3%	5.8 ± 0.3%

**Figure 2:**
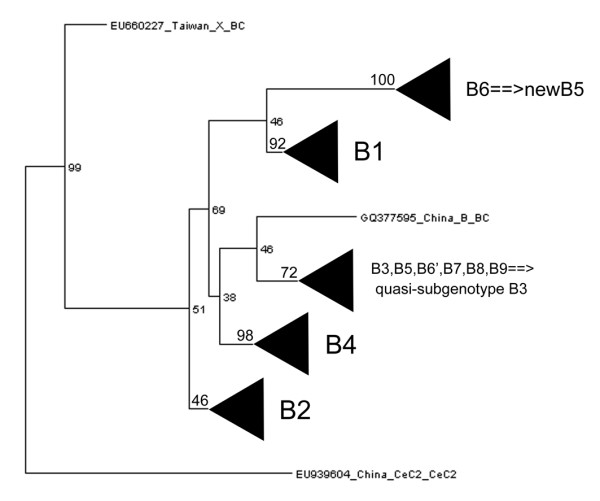
The novel classification of subgenotypes for genotype B HBV.

Taken together, we corrected the incongruence in the classification of genotype B HBV and proposed a consistent classification for genotype B based on a phylogenetic analysis of all genotype B HBV complete genome sequences (Figure 2). In this classification, originally designated B3, B5, B7, B8, B9 and B6’ (sequences from China) comprised a quasi-subgenotype B3. For continuous numbering, the subgenotype B6 has been renamed as new B5. This classification is well supported by both phylogenetic analysis and sequence divergences. To avoid misclassification of HBV subgenotypes, we insist that the rules [24,26] proposed to define novel genotypes and subgenotypes should be strictly obeyed. In addition, if possible, novel genotype/subgenotype should be made following a complete comparison of all relevant sequences rather than with a few representative sequences.

## Conclusions

Our results revealed that subgenotypes B1, B2, B4, and B6 (from Arctic) have been properly designated and should be maintained in the new classification. However, sequence divergences between previously designated B3, B5, B7, B8, B9 and another B6 (from China) were mostly less than 4%. In addition, subgenotype B3 did not form a monophyly. Therefore, current evidence failed to classify original B5, B7, B8, B9, and B6 (from China) as subgenotypes. Instead, they could be considered as a quasi-subgenotype B3 of Southeast Asian and Chinese origin. Moreover, previously designated B6 (from Arctic) should be renamed as B5 for continuous numbering. This novel classification is well supported by both the phylogeny and sequence divergence of > 4%.

## Abbreviations

HBV: Hepatitis B virus.

## Competing interests

The authors declare that they have no competing interests.

## Authors’ contributions

Conceived and designed the experiments: WS, ZZ. Performed the experiments: WS, CZ, WZ. Analyzed the data: WS. Wrote the paper: WS. Revised the paper: MJC, DGH and ZZ. All the authors read and approved the final manuscript.

## Pre-publication history

The pre-publication history for this paper can be accessed here:

http://www.biomedcentral.com/1471-230X/12/116/prepub

## Supplementary Material

Additional file 1 The phylogenetic tree constructed using all genotype B HBV sequences. Click here for file
