# Supplementary figures and images for: Subgenotype reclassification of genotype B hepatitis B virus
Source: BMC Gastroenterol. 2012 Aug 27;12:116. doi: 10.1186/1471-230X-12-116 (PMC3523008; doi:10.1186/1471-230X-12-116)

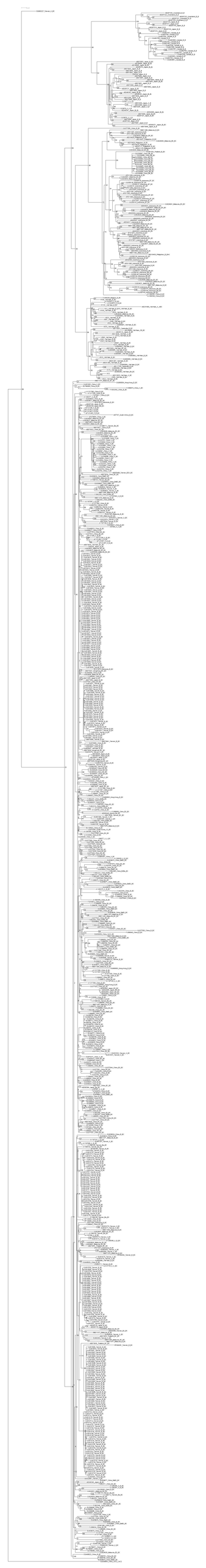

Supplement: Additional file 1 — The phylogenetic tree constructed using all genotype B HBV sequences. [file 1471-230X-12-116-S1.tiff]
